# *Wolbachia* and mosquitoes: Exploring transmission modes and coevolutionary dynamics in Shandong Province, China

**DOI:** 10.1371/journal.pntd.0011944

**Published:** 2024-09-12

**Authors:** Chuanhui Zang, Xuejun Wang, Yan Liu, Haifang Wang, Qintong Sun, Peng Cheng, Ye Zhang, Maoqing Gong, Hongmei Liu

**Affiliations:** 1 Digestive Disease Hospital of Shandong First Medical University, Shandong Institute of Parasitic Diseases Shandong First Medical University & Shandong Academy of Medical Sciences, Jining, China; 2 Shandong Center for Disease Control and Prevention, Jinan, China; Kenya Agricultural and Livestock Research Organization, KENYA

## Abstract

Vector-borne diseases leave a large footprint on global health. Notable culprits include West Nile virus (WNV), St. Louis encephalitis virus (SLEV), and Japanese encephalitis virus (JEV), all transmitted by *Culex* mosquitoes. Chemical insecticides have been widely used to reduce the spread of mosquito-borne diseases. Still, mosquitoes are becoming more and more resistant to most chemical insecticides which cause particular harm to the ecology. *Wolbachia* belongs to the family Ehrlichiaceae in the order Rickettsiales and is a matrilineally inherited endosymbiont present in 60% of insects in nature. *Wolbachia* is capable of inducing a wide range of reproductive abnormalities in its hosts, such as cytoplasmic incompatibility, and can alter mosquito resistance to pathogen infection. *Wolbachia* has been proposed as a biological alternative to chemical vector control, and specific research progress and effectiveness have been achieved. Despite the importance of *Wolbachia*, this strategy has not been tested in *Culex pipiens pallens*, the most prevalent mosquito species in Shandong Province, China. Little is known about how the mass release of *Wolbachia*-infected mosquitoes may impact the genetic structure of *Culex pipiens pallens*, and how the symbiotic bacterium *Wolbachia* interacts with mitochondria during host mosquito transmission. Based on the population genetic structure of *Culex pipiens pallens* in Shandong Province, this study investigated the infection rate and infection type of *Wolbachia* in Shandong Province and jointly analysed the evolutionary relationship between the host mosquito and the symbiotic bacterium *Wolbachia*. Our study showed that *Wolbachia* naturally infected by *Culex pipiens pallens* in Shandong Province was less homologous to *Wolbachia* infected by *Aedes albopictus* released from mosquito factory in Guangzhou. Our results also show that *Culex pipiens pallens* is undergoing demographic expansion in Shandong Province. The overall *Wolbachia* infection rate of *Culex pipiens pallens* was 92.8%, and a total of 15 WSP haplotypes were detected. We found that the genetic diversity of *Wolbachia* was low in *Culex pipiens pallens* from Shandong Province, and the mosquitoes were infected only with type B *Wolbachia*. Visualizing the relationship between *Culex pipiens pallens* and *Wolbachia* using a tanglegram revealed patterns of widespread associations. A specific coevolutionary relationship exists between the host mosquito and *Wolbachia*. Knowledge of this mosquito–*Wolbachia* relationship will provide essential scientific information required for *Wolbachia*-based vector control approaches in Shandong Province and will lead to a better understanding of the diversity and evolution of *Wolbachia* for its utility as a biocontrol agent.

## Author summary

This study was the first to investigate the population genetic structure and *Wolbachia* infection of *Culex pipiens pallens* in Shandong Province, providing a basis for mosquito-borne control and disease prevention. The joint analysis of *Wolbachia* and mitochondrial markers highlighted the coevolutionary relationship between *Wolbachia* and its host. It also provided the scientific basis for green precision prevention and control of mosquito-borne bacteria and viruses.

## Introduction

*Culex pipiens pallens*, the most abundant *Culex* mosquito species in northern China, is an essential vector for Bancroftian filariasis, Japanese encephalitis virus (JEV), and potentially West Nile virus(WNV) [1]. *Culex*-mediated disease outbreaks are increasing, posing a significant public health challenge [2]. Japanese encephalitis (JE) is a common viral encephalitis in Asia, with an annual incidence of 70,000 cases and 15,000 deaths; China accounts for 50% of the reported JE cases [3]. In 2018, the number of WNV infections spiked in 11 European Union/European Economic Area member states, with 1605 human cases, including 166 lethal cases [4]. Therefore, understanding the population genetic dynamics and genetic structure of *Cx*. *p*. *pallens* mosquitoes is essential to assessing their ability to transmit diseases and developing practical vector surveillance tools.

Shandong Province is located in eastern China along the lower reaches of the Yellow River, serving as a primary coastal province. The region exhibits a temperate monsoon climate that is often warm and rainy, making it well-suited to mosquito breeding. Historically, mosquito-borne infectious diseases such as malaria, JE, and filariasis have been prevalent in Shandong Province. For example, in the 1960s and 1970s, there were two major malaria outbreaks in Shandong Province, with more than 6 million and 4.6 million malaria cases, respectively [5]. In the early days, following the establishment of the People’s Republic of China in 1949, more than 5 million people were infected with microfilariae nationwide, and more than 2.5 million symptomatic patients were reported in Shandong Province [6]. Climate change may have accelerated the northward expansion of dengue outbreaks in China. Shandong Province is the northernmost focal point of dengue fever cases diagnosed in China [7], underscoring its epidemiological significance.

Mosquito control is essential for preventing vector-borne disease transmission in the human population. Insecticides are the primary weapons for mosquito control, and they have been extensively used to reduce the spread of mosquito-borne diseases, but with limited success. Meanwhile, mosquitoes are becoming increasingly insecticide-resistant due to the negligent and improper use of insecticides [8]. In light of these concerns, biological approaches are called upon as alternatives to chemical control [9]. For example, *Wolbachia* can reduce mosquito density and the spread of mosquito-borne diseases.

*Wolbachia* is a gram-negative symbiotic bacterium which is parasitic in invertebrates and can be transmitted through eggs. It is widely found in arthropods [10]. It is estimated that about 65% of insect species and 28% of mosquito species naturally carry *Wolbachia* [11]. The use of symbiotic *Wolbachia* for insect-borne disease control is primarily based on its ability to induce cytoplasmic incompatibility (CI) and resistance to pathogens. CI is a characteristic of *Wolbachia* infection that affects insect reproduction; eggs fertilized by a male carrying *Wolbachia* and laid by a female not carrying *Wolbachia*, or carrying a different type of *Wolbachia*, will not hatch [12,13]. *Wolbachia* can control mosquito populations using population suppression and population replacement [14]. In population suppression, male mosquitoes carrying *Wolbachia* are released into areas with uninfected or differently infected mosquitoes. This results in cytoplasmic incompatibility, leading to a decrease in the mosquito population. The targeted mosquito species can be eradicated by introducing many *Wolbachia*-infected male mosquitoes, leading to long-term infection. Another approach is to introduce female mosquitoes infected with a new *Wolbachia* strain, which resist the target mosquitoes, causing one-way cytoplasmic incompatibility. By releasing a sufficient number of these female mosquitoes and allowing them to multiply over several generations, *Wolbachia* can spread to all targeted mosquitoes [15]. This would result in replacing the original disease-transmitting mosquito vectors with new disease-resistant mosquito strains, thereby achieving the goal of interrupting disease transmission.

Mosquito vector control technology utilizing the symbiotic bacterium *Wolbachia* is a promising solution. Successful field trials across China [16], the United States [17], and Australia [18] have demonstrated superior efficiency, environmental protection, and sustainability. As an innovative approach to vector control, *Wolbachia* has been introduced into *Aedes albopictus* mosquitoes in Guangzhou, China, through microinjection, significantly suppressing the wild population by over 90%. The application of the insect symbiotic bacterium *Wolbachia* for mosquito vector control is based on its ability to induce CI. Controlling the *Wolbachia* type carried by the target population and establishing new *Wolbachia* mosquito strains are the basis of the application. However, it is unclear whether the mass release of *Wolbachia*-infected mosquitoes will impact other mosquito species, such as *Cx*. *p*. *pallens*. Understanding the effects of the mass release of *Wolbachia*-infected mosquitoes on the prevalence of *Wolbachia*, host population genetic structure, and the dynamics of gene flow patterns is crucial for assessing this population modification strategy’s long-term effectiveness and sustainability. Further research and surveillance are necessary to monitor any changes in *Wolbachia* prevalence and evaluate the impact of this intervention on disease transmission dynamics in Guangzhou and other areas implementing similar strategies.

Preparing incompatible vector insects often requires a lot of laboratory work, time, and technical expertise from the experimenter, who performs tasks such as removing the initially infected *Wolbachia* from the vector insect population and artificially infecting another type of *Wolbachia*, repeatedly backcrossing two vector insect populations (e.g., several generations of *Wolbachia*-infected and uninfected populations), and so on. The application of the resulting incompatible quins depends on the long-term stability of the new insect–*Wolbachia* symbiosis. Through long-term selection and evolution, natural populations are more stable than artificially created new mosquito populations. Therefore, whether natural populations can be directly used as incompatible insects for insect vector population control is worthy of further study.

Our research aimed to explore the following: i) Investigate *Wolbachia*’s infection rate and distribution pattern in *Cx*. *p*. *pallens* in Shandong Province and construct a phylogenetic tree for typing and tracing; ii) Utilize mitochondrial cytochrome c oxidase subunit I (COI) gene fragments to assess the population genetic structure and spread of *Cx*. *p*. *pallens* and predict disease risk; and iii) Examine the evolutionary relationship between *Wolbachia* bacteria and the mitochondria of *Cx*. *p*. *pallens* to understand *Wolbachia*’s contribution to shaping genetic diversity in *Cx*. *p*. *pallens*. This research will help to assess the roles of *Cx*. *p*. *pallens* and *Wolbachia* in disease transmission and develop more effective management strategies for controlling mosquito-borne diseases.

## Methods

### Sample collection and preparation

**Fig 1:**
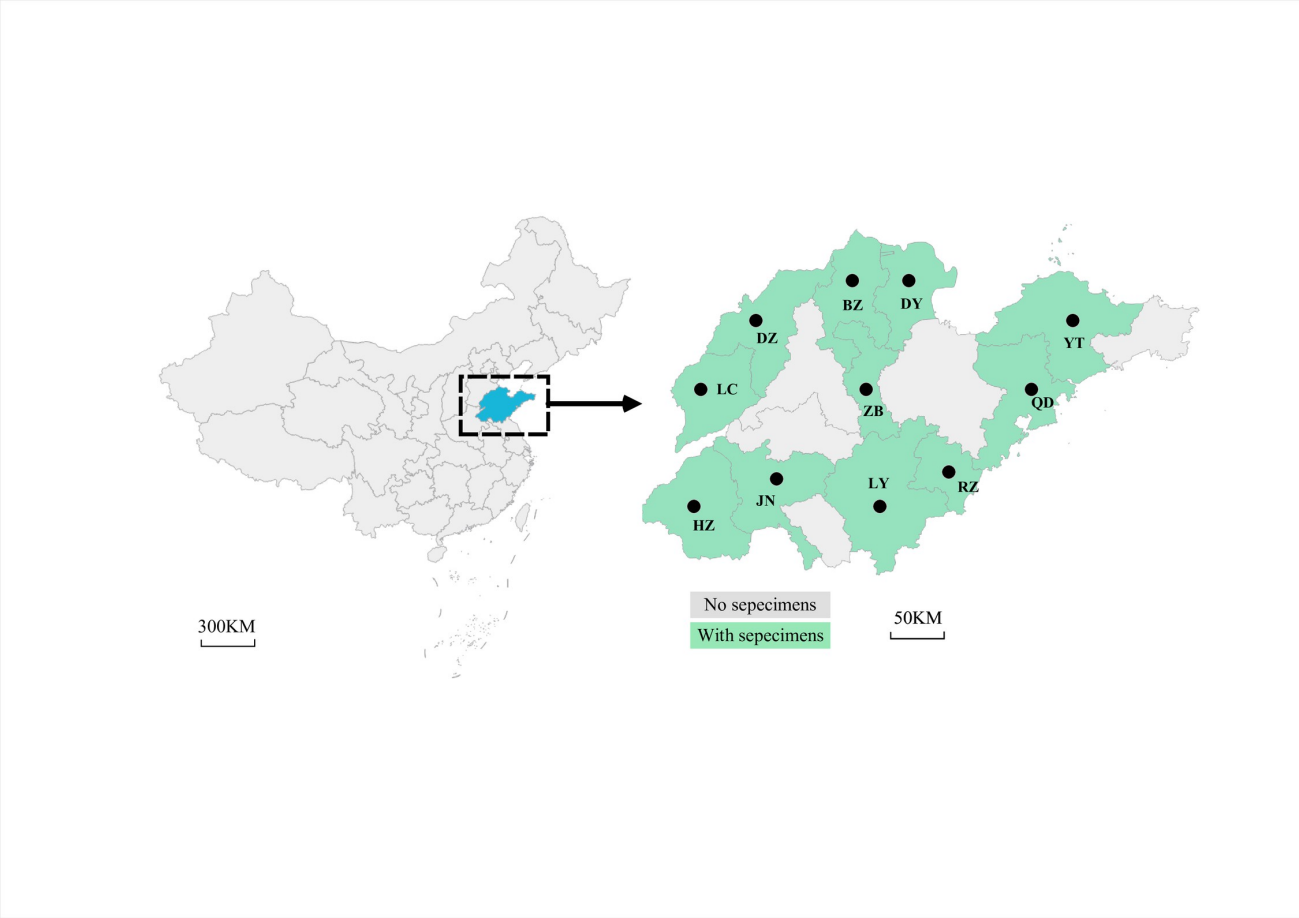
The distribution of field-collected *Culex pipiens pallens* sampled in this study. Site abbreviations: BZ–Binzhou; DZ–Dezhou; LC–Liaocheng; HZ–Heze; JN–Jining; DY–Dongying; ZB–Zibo; LY–Linyi; YT–Yantai; QD–Qingdao; RZ–Rizhao. The base layer of the map is from the Resource and Environment Science and Data Center (https://www.resdc.cn/).

*Culex p*. *pallens* were collected between July and September 2022 from 11 locations in Shandong Province (Fig 1 and Table 1). Captured mosquitoes were immediately preserved in separate sterile tubes containing 200 μl of RNAprotect Tissue Reagent. The samples were transported to the laboratory at room temperature for species identification and DNA extraction.

**Table 1:** Summary of *Culex pipiens pallens* specimen collection sites in Shandong Province.

Site ID	Latitude (N)	Longitude (E)	Collection date	Specimen genotyped	*Wolbachia* infection(%)
Northwest Shandong Plain					
Dezhou	37°45′	116°31′	18-Jul	115	99.1
Jiaolai Hills					
Yantai	37°54′	121°39′	13-Jul	170	100
Qingdao	36°09′	120°37′	28-Jun	56	87.5
Southwest Shandong Plain					
Liaocheng	36°46′	115°98′	4-Jul	69	92.7
Heze	35°25′	115°47′	18-Aug	137	98.5
Alluvial plain of the Yellow River					
Dongying	37°46′	118°49′	22-Aug	44	68.1
Binzhou	37°36′	118°03′	5-Aug	93	96.7
South-central Shandong Plain					
Zibo	36°81′	118°04′	12-Jun	28	96.4
Linyi	35°07′	118°33′	11-Aug	50	86
Rizhao	35°42′	119°46′	15-Jul	59	64.4
Jining	36°40′	117°02′	9-Jun	70	97.1

### Mosquito species identification and DNA extraction

Mosquito species were identified microscopically based on their morphological characteristics (https://www.wrbu.si.edu/) (Olympus, SZX7, Japan) and further confirmed using molecular marker analysis. Molecular identification was achieved with COI and internal transcribed spacer 2 (ITS2) barcoding, using BLAST (https://blast.ncbi.nlm.nih.gov/Blast.cgi) to determine sequence percentage identity. DNA was extracted from individual mosquitoes using the cador Pathogen 96 QIAcube HT Kit following the manufacturer’s directions, and stored at -80°C.

### PCR identification of Wolbachia infections

Detection of the *Wolbachia* endosymbiont in mosquitoes was performed using PCR-based molecular approaches using the most common *Wolbachia*-specific DNA marker (the WSP gene), with the forward primer 81F (5′-TGGTCCAATAAGTGATGAAGAAAC-3′) and reverse primer 691R (5′-AAAAATTAAACGCTACTCCA-3′) [19]. PCR reaction mixtures comprised 25 μl of 2× Phanta Max Master Mix, 1 μl each of 10-μM forward and reverse primers, 2 μl of template DNA, and nuclease-free water to a final volume of 50 μl. PCR conditions were as follows: 95°C for 2 min; 35 cycles of 95°C for 30 s, 54°C for 45 s, and 72°C for 1 min; and 72°C for 5 min. PCR products were separated electrophoretically and sequenced at Sangon Biotech (Shanghai, China).

### Amplification and sequencing of the COI gene

The COI gene of the mitochondrial genome was amplified using primers LCO 1490 (5′-GGTCAACAAATCATAAAGATATTGG-3′) and HCO 2198 (5′-TAAACTTCAGGGTGACCAAAAAATCA-3′) to determine population genetic structure [20]. PCR reaction mixtures comprised 25 μl of 2× Phanta Max Master Mix, 1 μl each of 10-μM forward and reverse primers, 2 μl of template DNA, and nuclease-free water to a final volume of 50 μl. PCR conditions were as follows: 94°C for 1 min; 5 cycles of 94°C for 40 s, 45°C for 40 s, and 72°C for 1 min; 30 cycles of 94°C for 40 s, 53°C for 40 s, and 72°C for 1 min; and 72°C for 5 min. The PCR products were sequenced by Sangon Biotech (Shanghai, China).

### Data analysis

All sequences were manually aligned, checked and edited using BioEdit version 7.0 and compared with other sequences available in the GenBank database to determine the percentage identity using BLAST. The most similar sequences were downloaded for phylogenetic analysis. Specimens showing more than 99% nucleotide sequence identity with the available species sequences in the database were considered *Cx*. *p*. *pallens*. Based on the mitochondrial COI gene sequences, we used Arlequin v3.5 [21] and DnaSP v6 [22] to calculate the number of segregating sites (*s*), haplotypes (*h*), haplotype diversity (*Hd*), and nucleotide diversity (*pi*), and neutrality tests [23], namely Tajima’s *D* test and Fu’s *Fs* test, to investigate the genetic diversity of *Cx*. *p*. *pallens* [24]. To determine the genetic structure of the mosquito populations, we used analysis of molecular variance (AMOVA) to partition the genetic variation among groups (*Fct*), populations within groups (*Fsc*), and populations among groups (*Fst*) [25]. Pairwise *Fst* and gene flow (*Nm*) values were calculated for all the populations. In addition, haplotype networks of *Cx*. *p*. *pallens* were created using the haplotypes network (TCS) method in PopART 1.7 to visualise the relationships among populations. Geographical population structure was evaluated using the Bayesian clustering method in STRUCTURE v.2.3, which identifies the most probable number of genetic clusters (*K*) and assigns individuals to these clusters. Different runs were conducted using different datasets for further clustering. Subsequently, *COI* and *WSP* sequences were aligned using ClustalX, and a neighbour-joining phylogenetic tree model based on genetic distance values was created using MEGA X software [26].

## Results

### Prevalence and genotyping of Wolbachia in wild-caught mosquitoes

**Fig 2:**
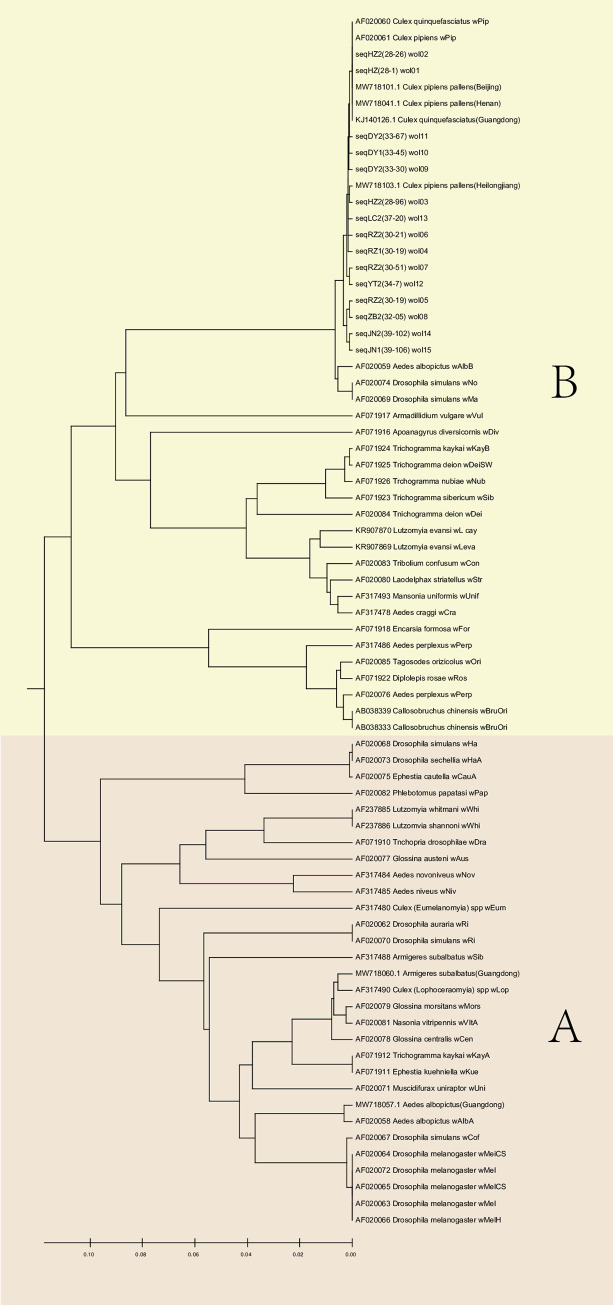
Maximum likelihood phylogenetic tree based on surface protein gene sequences for *Wolbachia* from different hosts. Sequences extracted from GenBank.

All adult *Cx*. *p*. *pallens* mosquitoes collected from the 11 localities in Shandong Province were examined for *Wolbachia* infection based on the presence or absence of the WSP gene. Overall, the infection prevalence was high, with 827 of 891 (92.8%) individuals being positive for *Wolbachia*. Notably, *Wolbachia* infection rates varied among cities, with the lowest (64.4%) in Rizhao and the highest (100%) in Yantai (S1 Fig). Fifteen WSP haplotypes were detected in *Wolbachia* infections and clustered into 15 putative strains, named Wol 01 to Wol 15. Zhou et al. [19] classified all WSP sequences into two groups of *Wolbachia* strains, corresponding to types A and B. In this study, all the *Wolbachia* infections in *Cx*. *p*. *pallens* were type B infections (Fig 2).

### Polymorphism of the mitochondrial gene COI sequence

**Fig 3:**
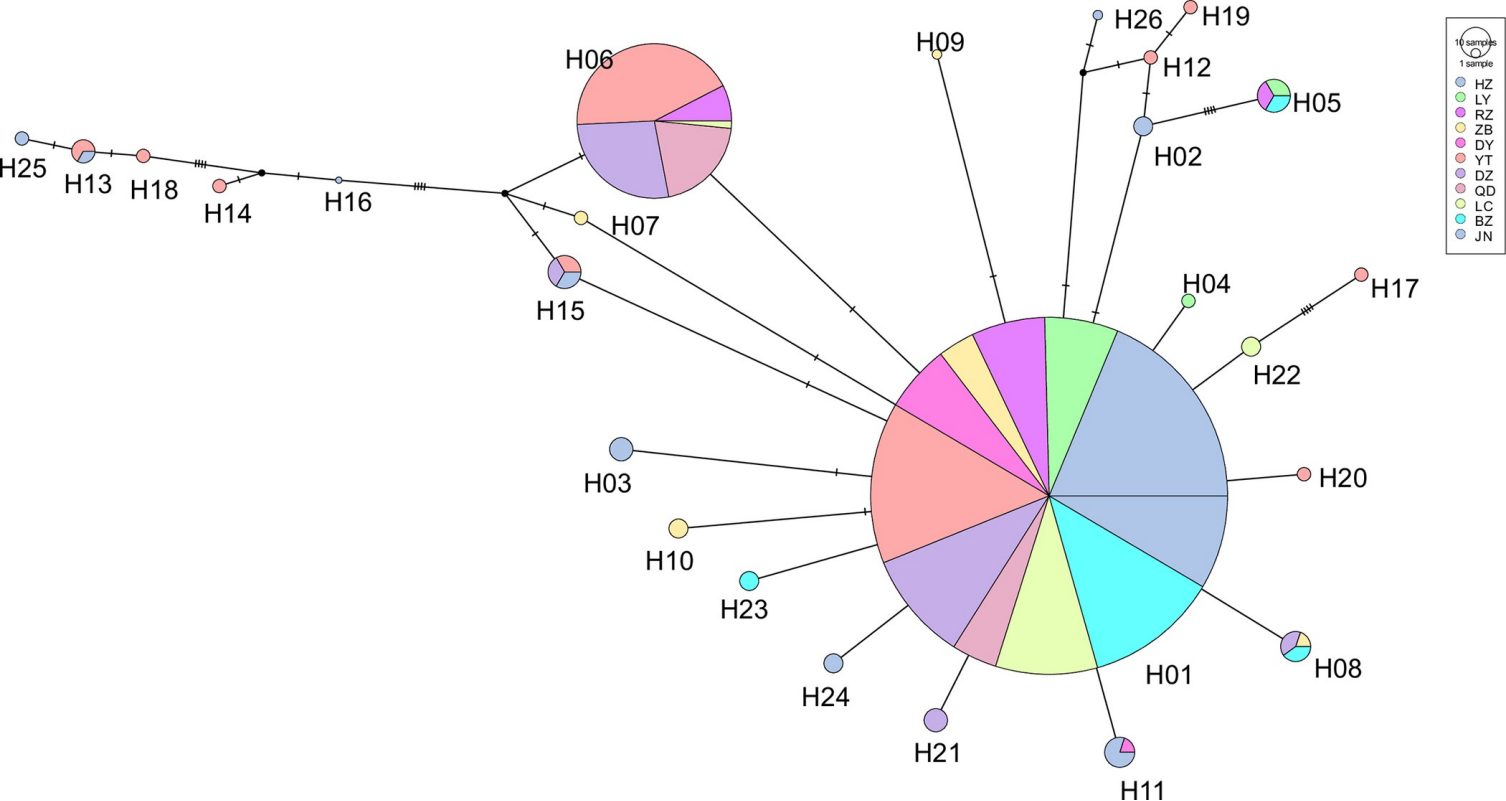
Phylogenetic network of 11 mitochondrial haplotypes of the cytochrome oxidase subunit I gene in *Culex pipiens pallens* in Shandong Province.

A total of 1782 COI sequences were generated from the 11 populations. The COI sequences were aligned, yielding a total length of 603 bp and 27 variable sites. The overall haplotype diversity (*Hd*) and nucleotide diversity (*Pi*) were 0.352 and 0.98 × 10^˗2^, respectively (Table 2). Among the 11 populations, *Cx*. *p*. *pallens* from Dezhou exhibited the highest haplotype diversity, and that from Yantai exhibited the highest nucleotide diversity (Table 2). In general, *Cx*. *p*. *pallens* from coastal cities exhibited higher COI haplotype and nucleotide diversity than those from other cities. Based on differences in the nucleotide composition of the COI gene, we identified 26 mitochondrial haplotypes in the 11 studied populations, which were denoted as H01–H26 (Fig 3).

**Table 2:** Polymorphism of COI and neutrality tests of *Culex pipiens pallens* populations.

Site	n	s	*Pi* (*10^−2^)	h	Hd	Tajima’s *D*	p	Fu’s *Fs*	p
Northwest Shandong Plain									
Dezhou	230	5	0.097	6	0.532	˗0.55576	0.35800	˗1.40882	0.30900
Jiaolai Hills									
Yantai	340	13	0.187	10	0.522	˗1.03730	0.14500	˗1.81529	0.25400
Qingdao	112	1	0.084	2	0.504	1.86793	0.98400	2.41107	0.83800
Southwest Shandong Plain									
Liaocheng	138	2	0.019	3	0.112	˗0.99803	0.09800	-1.98235	0.07400
Heze	274	2	0.012	3	0.071	˗1.01339	0.10200	˗2.58453	0.05300
Alluvial plain of the Yellow River									
Dongying	88	1	0.007	2	0.045	˗0.91012	0.17300	˗1.37585**	0.00560
Binzhou	186	8	0.053	5	0.145	˗1.71164*	0.01100	˗2.33474	8.15100
South-central Shandong Plain									
Zibo	56	4	0.052	5	0.293	-1.42345	0.05900	˗3.28950**	0.00500
Linyi	100	6	0.071	3	0.116	-1.41815	0.06200	0.41333	0.53400
Rizhao	118	6	0.102	3	0.338	-0.99169	0.16200	1.35630	0.75400
Jining	140	16	0.152	8	0.298	-1.78621*	0.01400	˗1.29182	8.29988

*P < 0.05

**P < 0.01

*n* = number of genes (two per individual), *s* = number of polymorphic (i.e., segregating) sites, *Pi* = nucleotide diversity, *h* = number of haplotypes, *Hd* = haplotype diversity.

A haplotype network graph was constructed using the TCS method. The size of each circle is proportional to its corresponding frequency. Different colours indicate different populations. The number of small diagonal lines refer to variable asynchronous numbers.

### Haplotype network reconstruction of Cx. p. pallens

The statistical parsimony network was generated with TCS using COI sequences of *Cx*. *p*. *pallens*, identifying 26 haplotypes across the sampled sites (Fig 3). H01 and H06 were the most frequently observed haplotypes. H01 was the central haplotype that was highly connected to the haplotype lines and was the only haplotype found in all the localities in Shandong Province. Nearly all other haplotypes originated from H01 through one or more mutations. The haplotype distribution showed that H01, H05, H06, H08, H11, H13 and H15 were shared among multiple populations, whereas the remaining haplotypes were only observed in a single population.

### Population genetic structure

A total of 891 individuals from 11 populations were analysed for population genetic diversity. Pairwise comparisons indicated that *Fst* values were significantly different from zero, with the lowest value (0.00109) found between Binzhou and Linyi and the highest value (0.54103) between Qingdao and Heze (Table 3). Generally, highly significant pairwise population differentiation was observed between Qingdao and most other populations (*Fst* > 0.25). However, frequent gene flow occurred in other populations (*Nm* > 1). The AMOVA showed that the majority of genetic variance occurred within populations (87.91%) (Table 4). The total *Fst* was 0.13135 (*P* > 0.05) and *Nm* was 1.65, reflecting moderate population differentiation.

**Table 3:** Mitochondrial DNA-based population differentiation for population pairs (estimates of *Fst* below the diagonal and *Nm* above the diagonal).

	DY	BZ	DZ	HZ	LC	LY	QD	RZ	YT	JN	ZB
DY	-	16.33	0.80	17.75	12.93	9.05	0.3	2.56	1.13	10.88	8.16
BZ	0.01568	-	1.13	19.90	14.25	664.76	0.64	4.31	1.38	10.52	12.76
DZ	0.23808	0.18052	-	0.83	1.07	1.21	5.11	6.23	26.27	1.73	1.10
HZ	0.01389	0.01241	0.23112	-	12.65	11.04	0.31	2.73	1.16	9.59	8.32
LC	0.01897	0.01724	0.16888	0.01938	-	8.98	0.38	3.90	1.43	9.47	8.21
LY	0.02688	0.00038	0.17107	0.02215	0.02710	-	0.49	5.30	1.46	9.81	8.06
QD	0.45650	0.36122	0.04664	0.44496	0.39740	0.33679	-	1.42	8.20	0.73	0.43
RZ	0.08883	0.05481	0.03855	0.08393	0.06022	0.04505	0.14999	-	5.19	4.44	3.12
YT	0.18080	0.15300	0.00943	0.17703	0.14897	0.14659	0.02958	0.04593	-	2.12	1.36
JN	0.02245	0.02321	0.12643	0.02540	0.02572	0.02486	0.25522	0.05330	0.10542	-	9.41
ZB	0.02974	0.01922	0.18567	0.02919	0.02956	0.03009	0.36619	0.07417	0.15531	0.02588	-

DY: Dongying; BZ: Binzhou; DZ: Dezhou; HZ: Heze; LC: Liaocheng; LY: Linyi; QD: Qingdao; RZ: Rizhao; YT: Yantai; JN: Jining; ZB: Zibo

**Table 4:** Analysis of molecular variance test of all 11 *Culex pipiens pallens* populations collected from different regions of Shandong Province.

Source of variation	d.f.	Sum of squares	Variance components	Percentage of variation
Among populations	10	59.331	0.03597	12.09
Within populations	1770	462.799	0.26147	87.91

The Fu’s *Fs* and Tajima’s *D* values for the *Cx*. *p*. *pallens* populations in Shandong Province were almost entirely negative, except in Qingdao (Table 2). Tajima’s *D* value (˗1.97) and Fu’s *Fs* value (˗27.58) for the overall population were significant, suggesting a high number of low-frequency mutations and a demographic expansion of *Cx*. *p*. *pallens* in Shandong Province. Among specific populations, Jining and Binzhou had significantly negative *D* values, whereas Dongying and Zibo exhibited significant negative *Fs* values. Tajima’s *D* and Fu’s *Fs* tests revealed that Jining, Binzhou, Dongying, and Zibo were significantly negative, suggesting recent population expansion or selection.

Multilocus cluster Bayesian analysis of all 11 population samples indicated the genetic structure among *Cx*. *p*. *pallens* populations (*K* = 2–11) (S2A Fig) [27]. The results show that when *K* = 4, a larger Delta *K* value is obtained, and when *K* = 7, the Delta *K* value is the largest. Therefore, the optimal *K* value should be 4–7 (S2B Fig). Based on the geographical distribution and genetic differentiation within Shandong Province, we divided the 11 populations into five groups: the Northwest Shandong Plain (Dezhou), the Jiaolai Hills (Yantai, Qingdao), the Southwest Shandong Plain (Liaocheng, Heze), the alluvial plain of the Yellow River (Dongying, Binzhou), and the south-central Shandong Plain (Zibo, Linyi, Rizhao, Jining).

### Migration and gene flow patterns

**Fig 4:**
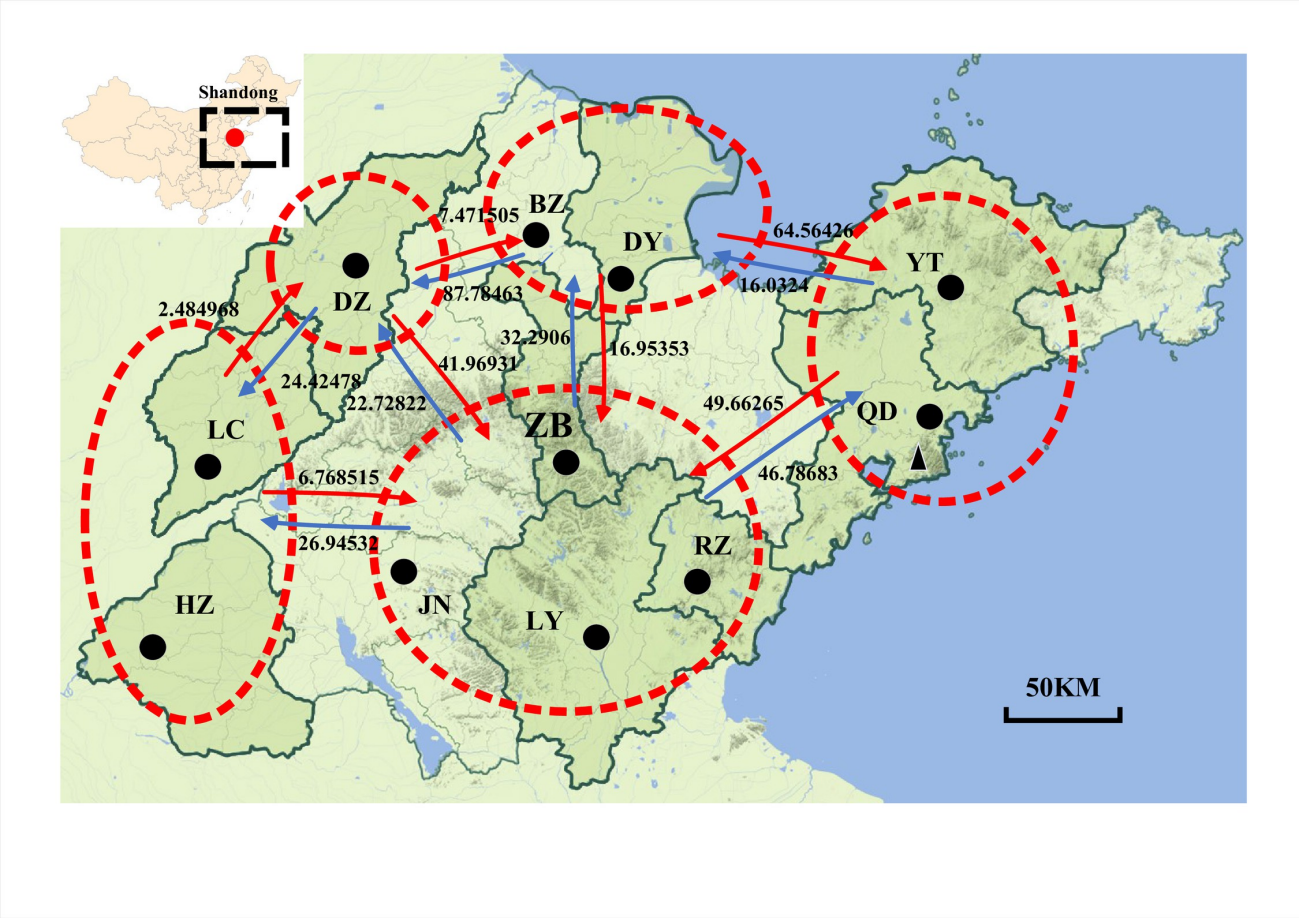
Bayesian estimates of historical asymmetrical migration between populations of *Culex pipiens pallens*. The five locality groups are indicated by dotted circles, and arrows indicate the direction of migration. The base layer of the map is from the Resource and Environment Science and Data Center (https://www.resdc.cn/).

LAMARC analysis revealed that historical gene flow rates ranged from 0.88 to 96.02. The highest migration rates were detected among neighbouring populations within each of the five locality groups (Fig 4). Moderate migration levels were observed in the five locality groups. Based on the coalescent analysis, migration was asymmetrical (Fig 4).

### Evolutionary relationships between mosquitoes and Wolbachia

We recorded mosquito–*Wolbachia* associations in wild-caught mosquitoes from Shandong Province. Visualisation of these associations using a tanglegram revealed patterns of broad associations (S3 Fig). The distance-based quantitative test showed no significant consistency between the mosquito and *Wolbachia* phylogenies at the global level (ParaFit Global test: ParaFit Global < 0.001, *P* = 1). Among the host–endosymbiont links, the associations between H07 and Wol 08, H07 and Wol 12, H07 and Wol 13, and H15 and Wol07 were statistically significant (Table 5).

**Table 5:** Results of the ParaFit analysis for *Culex pipiens pallens* and *Wolbachia*.

Host	Wolbachia	PF1.statistic	PF1 *P*-value	PF2.statistic	PF2 *P*-value
H07	Wol08	˗3.79×10^−9^	0.03	-1.5×10^−3^	0.03
H07	Wol12	˗5.07×10^−9^	0.04	-2.01×10^−3^	0.04
H07	Wol13	˗1.92×10^−9^	0.03	-0.76×10^−3^	0.03
H15	Wol07	˗2.77×10^−9^	0.04	-1.1×10^−3^	0.04

## Discussion

### Detection of Wolbachia infection and its distribution in wild mosquitoes

In this study, we assessed the prevalence of *Wolbachia* in *Cx*. *p*. *pallens* collected from Shandong Province. The overall infection rate of all tested mosquitoes was 92.8%, indicating that *Wolbachia* was widespread in *Cx*. *p*. *pallens*.

The WSP molecular marker enabled successful detection of *Wolbachia* infection across numerous taxa, strain genotyping, and evolutionary comparison between the detected *Wolbachia* strains. In this study, a low genetic diversity of *Wolbachia* strains was found in *Cx*. *p*. *pallens*, and natural populations were infected only with type B *Wolbachia*, not with type A. Studies have shown that animal habitats such as wetlands and forests tend to have relatively high infection rates of mosquito-borne viruses and bacteria [28]. However, *Wolbachia* infection rates of *Cx*. *p*. *pallens* in the Yellow River Delta wetland of Dongying, an important habitat for migratory birds [29], were lower than those in other places. This presents a new opportunity for the application of *Wolbachia* to mediate the transmission of mosquito-borne diseases by *Cx*. *p*. *pallens*.

### The impact of the Guangzhou“mosquito factory” on mosquitoes in other areas

The centrepiece of the “mosquito factory” is a colony of *Wolbachia* mosquitoes, called the brood stock, from which all future populations of *Wolbachia* mosquito offspring are bred. Owing to the role of cytoplasmic incompatibility, the release of *Wolbachia*-infected male mosquitoes into the wild has become a promising strategy for suppressing wild mosquito populations [30]. However, more attention should be paid to the effects of *Wolbachia* on hosts’ behavioural patterns to evaluate the environmental impact of releasing these *Wolbachia*-infected insects into the field [31]. Notably, a large number of *Aedes albopictus* infected with *Wolbachia* were released in Guangzhou. So, will the increase or decrease in the abundance of the target species lead to the disruption of the original ecological balance. It is generally agreed that the success of *Wolbachia* is due to its ability to switch from one host species to another [32]. This raises further questions about its effects on other species.

The population of *Cx*. *p*. *pallens* is expanding in Shandong Province. We believe that apart from assessing the geographic regions where climate change, the taxa of hosts and transmission types of pathogens, future research should also consider the potential effects of *Wolbachia* more carefully. Finally, we sought to inform future research avenues, policy, and practices via the trends and impacts identified herein.

In the present study, we found that the *Wolbachia* infection rate of *Cx*. *p*. *pallens* in Shandong Province is high, and phylogenetic tree analysis shows that it has low homology with *Wolbachia* infection of *Aedes albopictus* in Guangzhou, but high homology with *Wolbachia* infection of *Culex* mosquitoes in Guangzhou. Questions about the influence of *Wolbachia* infection on host behaviour under field conditions, and consequently on the ecosystem, remain to be addressed. The molecular mechanisms by which *Wolbachia* affect host behaviour also need to be elucidated in more detail. The effects of *Wolbachia* on host fitness traits may be multidimensional, and a number of host genes, miRNAs and proteins may be modified by *Wolbachia* infection.

### Geographical isolation of Cx. p. pallens populations from Qingdao

In the present study, we analysed the population genetics of *Cx*. *p*. *pallens* collected from different regions of Shandong Province based on the COI gene. Most (87.91%) of the genetic variation occurred within individuals, whereas only 12.09% of the variation was detected among populations. Our data showed significant genetic differentiation and limited gene flow between the Qingdao population and populations from most other cities. In contrast to other cities within Shandong Province, Qingdao is primarily situated on hilly terrain that slopes from east (Laoshan Mountain Group) to west (Jiaozhou Bay), with rolling hills in the north and the Fushan Mountain Range to the south along the Pacific coast. The expansive Pacific Ocean thus forms a natural geographical barrier between Qingdao and other cities within the province. In addition to Qingdao, other cities also have varying geographical environments. Therefore, precise countermeasures should be adopted for mosquito vector prevention and control.

### Demographic expansion of Cx. p. pallens in Shandong Province

The demographic expansion of *Cx*. *p*. *pallens* closely corresponded to the COI haplotype network. The haplotype profiles were star-shaped, reflecting their recent appearance and rapid population growth. The neutrality test results were significantly negative, which also supports this phenomenon. A strong link exists between demographic expansion and climatic variability. Meteorological factors, including temperature, humidity, and precipitation, have a significant impact on the number, density, and distribution of disease vectors, as well as the spatial and temporal dynamics, epidemic frequency, and intensity of vector-borne diseases. In addition, cluster analysis revealed that *Cx*. *p*. *pallens* community structure existed spatial and temporal heterogeneity to some extent. Mosquito development and survival and viral replication depend on environmental conditions, particularly climatic conditions. A study has shown that an increase in temperature was observed from 1970 to 2021 in most places in China, and annual change rates varied substantially among different sites, from ˗0.22°C/year to 0.58°C/year [7]. Notably, Shandong Province reported an autochthonous dengue case for the first time on 16 August 2017. Ninety-five cases were subsequently reported across the entire province, and 79 indigenous cases occurred in Jining, the northernmost region where local dengue fever cases have been detected [33]. Therefore, upgrading surveillance systems for vectors and vector-borne diseases in the context of climate change will strengthen research on risk assessments, predictions, early warnings, control strategies, and intervention measures to effectively cope with the new challenges of climate-sensitive vector-borne diseases.

### Evolutionary relationships between mosquitoes and Wolbachia

Symbiosis is a major force of evolutionary change that influences virtually all aspects of biology, from population ecology and evolution to genomics and molecular/biochemical mechanisms of development and reproduction. A broad association pattern between mosquitoes and *Wolbachia* strains was observed based on the tanglegram (S3 Fig). A previous study reported that *Aedes* mosquitoes tended to be associated with *Wolbachia* supergroup A, whereas other mosquito species, particularly those of the genus *Culex*, were largely associated with *Wolbachia* supergroup B. This indicated that closely related *Wolbachia* strains are likely to establish themselves in related hosts. In the present study, we found that all *Wolbachia* infections in *Cx*. *p*. *pallens* were associated with *Wolbachia* supergroup B, which is consistent with the above results. Significant coevolution was detected between Wol08, Wol12, and Wol13 and haplotype H07, and between Wol07 and haplotype H15. These regional variations in mosquito–*Wolbachia* interactions may represent an ongoing evolutionary process, or infections may occur by chance or be associated with local environments. Therefore, further investigation is warranted. Understanding *Wolbachia* host specificity has significant implications, particularly for optimising *Wolbachia* biocontrol strategies. In addition to selecting strains that can effectively limit pathogen replication, strains should also be selected based on their host specificity.

This study has potential limitations, including a) differences in sampling location and environment, which may lead to potential sample size bias and not fully represent the actual genetic diversity of the population; and b) the existence of a specific coevolutionary relationship between the haplotype of some host mosquitoes and the symbiotic *Wolbachia*. The significance of this relationship for precise control should be studied in the next step.

## Conclusions

Based on the results of population genetic structure and *Wolbachia* infection, this study has provided valuable insights for controlling *Cx*. *p*. *pallens* in Shandong Province. From this analysis, it is evident that the population of *Cx*. *p*. *pallens* is expanding, increasing the risk of human exposure to vector mosquitoes and infection with arboviruses, and thus the need to strengthen preventive and early warning measures for related diseases. Additionally, the geographical isolation between the Qingdao population and the majority of populations in Shandong Province suggests that more precise control measures should be implemented based on local conditions. Phylogenetic analysis showed low homology between *Wolbachia* strains infecting *Aedes albopictus* released from mosquito factories in Guangzhou and *Wolbachia* strains naturally infecting *Cx*. *p*. *pallens*. This finding underscores the importance of understanding *Wolbachia*’s non-changing host characteristics for future mosquito control strategies. The investigation based on the co-evolutionary relationship between *Wolbachia* and *Cx*. *p*. *pallens* mitochondrial markers suggests that the host specificity of *Wolbachia* is crucial in optimizing its biocontrol strategy. Therefore, in addition to considering strains that can effectively limit pathogen replication, the selection of strains should be based on host specificity to implement biological control. This study provides a valuable reference for the scientific and accurate control of mosquito-borne diseases in the future.

## Supporting information

S1 Data Fifteen WSP haplotypes sequence were detected in Wolbachia infections and named Wol 01 to Wol 15. (DOCX)

S1 Fig *Wolbachia* prevalence in 11 populations of *Culex pipiens pallens* in Shandong Province based on PCR amplification of the WSP marker. The prevalence in Rizhao and Dongying was 64.10%-69.20%; in Qingdao and Linyi, 69.21%-88.20%; in Liaocheng, 88.21%-92.70%; and in Dezhou, Yantai, Heze, Binzhou, Zibo and Jining, 92.715%-100.00%. The base layer of the map is from the Resource and Environment Science and Data Center (https://www.resdc.cn/).
(TIF)

S2 Fig Population structure of *Culex pipiens pallens* in Shandong Province. **A.** Stacked bar plots of STRUCTURE for K = 2 to 11 subgroups. Each individual is represented by a vertical bar, partitioned into coloured segments with the length of each segment representing the proportion of the individual’s genome; **B.** Delta K plotted against putative K ranging from 2 to 11.
(TIF)

S3 Fig Tanglegram of mosquito cytochrome oxidase subunit I neighbour-joining tree compared to the *Wolbachia* endosymbiont neighbour-joining tree. Lines indicate the host–endosymbiont association that was significant in the Global ParaFit test of congruence between host and endosymbiont phylogenies.
(TIF)
