# Supplementary material for: Wolbachia and mosquitoes: Exploring transmission modes and coevolutionary dynamics in Shandong Province, China
Source: PLoS Negl Trop Dis. 2024 Sep 12;18(9):e0011944. doi: 10.1371/journal.pntd.0011944 (PMC11421781; doi:10.1371/journal.pntd.0011944)
Supplement: S1 Data — (DOCX) [file pntd.0011944.s001.docx]

>Wol 01

CAAGAATTGACGGCATTGAATATAAAAAAGGAACCGAAGTTCATGATCCTTTAAAAGCAT

CTTTTATGGCTGGTGGTGCTGCATTTGGTTATAAAATGGACGATATCAGGGTTGATGTTG

AGGGACTTTACTCACAACTAAACAAAAACGACGTTAGTGGTGCAACATTTACTCCAACAA

CTGTTGCAAACAGTGTGGCAGCATTTTCAGGATTGGTTAACGTTTATTACGATATAGCGA

TTGAAGATATGCCTATCACTCCATACGTTGGTGTTGGTGTTGGTGCAGCATATATCAGCA

ATCCTTCAGAAGCTAGTGCAGTTAAAGATCAAAAAGGATTTGGTTTTGCTTATCAAGCAA

AAGCTGGTGTTAGTTATGATGTAACCCCAGAAATCAAACTCTTTGCTGGTGCTCGTTATT

TTGGTTCTTATGGTGCTAGTTTTAATAAAGAAGCAGTATCAGCTACTAAAGAGATCAATG

TCCTTTACAGCGCTGTTGGTGCAGAAGCTGGAGT

>Wol 02

CAAGAATTGACGGCATTGAATATAAAAAAGGAACCGAAGTTCATGATCCTTTAAAAGCAT

CTTTTATGGCTGGTGGTGCTGCATTTGGTTATAAAATGGACGATATCAGGGTTGATGTTG

AGGGACTTTACTCACAACTAAACAAAAACGACGTTAGTGGTGCAACATTTACTCCAACAA

CTGTTGCAAACAGTGTGGCAGCATTTTCAGGATTGGTTAACGTTTATTACGATATAGCGA

TTGAAGATATGCCTATCACTCCATACGTTGGTGTTGGTGTTGGTGCAGCATATATCAGCA

ATCCTTCAGAAGCTAGTGCAGTTAAAGATCAAAAAGGATTTGGTTTTGCTTATCAAGCAA

AAGCTGGTGTTAGTTATGATGTAACCCCAGAAATCAAACTCTTTGCTGGTGCTCGTTATT

TTGGTTCTTATGGTGCTAGTTTTAATAAAGAAGCAGTATCAGCTACTAAAGAGATCAATG

TCCTTTACAGCGCTGTTGGTGCAGAAGCTGGAGT

>Wol 03

CAAGAATTGACGGCATTGAATATAAAAAAGGAACCGAAGTTCATGATCCTTTAAAAGCAT

CTTTTATGGCTGGTGGTGCTGCATTTGGTTATAAAATGGACGATATCAGGGTTGATGTTG

AGGGACTTTACTCACAACTAAACAAAAACGACGTTAGTGGTGCAACATTTACTCCAACAA

CTGTTGCAAACAGGGTGGCAGCATTTTCAGGATTGGTTAACGTTTATTACGATATAGCGA

TTGAAGATATGCCTATCACTCCATACGTTGGTGTTGGTGTTGGTGCAGCATATATCAGCA

ATCCTTCAGAAGCTAGTGCAGTTAAAGATCAAAAAGGATTTGGTTTTGCTTATCAAGCAA

AAGCTGGTGTTAGTTATGATGTAACCCCAGAAATCAAACTCTTTGCTGGTGCTCGTTATT

TTGGTTCTTATGGTGCTAGTTTTAATAAAGAAGCAGTATCAGCTACTAAAGAGATCAATG

TCCTTTACAGCGCTGTTGGTGCAGAAGCTGGAGT

>Wol 4

CAAGAATTGACGGCATTGAATATAAAAAAGGAACCGAAGTTCATGATCCTTTAAAAGCAT

CTTTTATGGCTGGTGGGGCTGCATTTGGTTATAAAATGGACGATATCAGGGTTGATGTTG

AGGGACTTTACTCACAACTAAACAAAAACGACGTTAGTGGTGCAACATTTACTCCAACAA

CTGTTGCAAACAGTGTGGCAGCATTTTCAGGATTGGTTAACGTTTATTACGATATAGCGA

TTGAAGATATGCCTATCACTCCATACGTTGGTGTTGGTGTTGGTGCAGCATATATCAGCA

ATCCTTCAGAAGCTAGTGCAGTTAAAGATCAAAAAGGATTTGGTTTTGCTTATCAAGCAA

AAGCTGGTGTTAGTTATGATGTAACCCCAGAAATCAAACTCTTTGCTGGTGCTCGTTATT

TTGGTTCTTATGGTGCTAGTTTTAATAAAGAAGCAGTATCAGCTACTAAAGAGATCAATG

TCCTTTACAGCGCTGTTGGTGCAGAAGCTGGAGT

>Wol 05

CAAGAATTGACGGCATTGAATATAAAAAAGGAACCGAAGTTCATGATCCTTTAAAAGCAT

CTTTTATGGCTGGGGGTGCTGCATTTGGTTATAAAATGGACGATATCAGGGTTGATGTTG

AGGGACTTTACTCACAACTAAACAAAAACGACGTTAGTGGTGCAACATTTACTCCAACAA

CTGGTGCAAACAGTGGGGCAGCATTTTCAGGATTGGTTAACGTTTATTACGATATAGCGA

TTGAAGATATGCCTATCACTCCATACGTTGGTGTTGGTGTTGGTGCAGCATATATCAGCA

ATCCTTCAGAAGCTAGTGCAGTTAAAGATCAAAAAGGATTTGGTTTTGCTTATCAAGCAA

AAGCTGGTGTTAGTTATGATGTAACCCCAGAAATCAAACTCTTTGCTGGTGCTCGTTATT

TTGGTTCTTATGGTGCTAGTTTTAATAAAGAAGCAGTATCAGCTACTAAAGAGATCAATG

TCCTTTACAGCGCTGTTGGTGCAGAAGCTGGAGT

>Wol 06

CAAGAATTGACGGCATTGAATATAAAAAAGGAACCGAAGTTCATGATCCTTTAAAAGCAT

CTTTTATGGCTGGTGGTGCTGCATTTGGTTATAAAATGGACGATATCAGGGTTGATGTTG

AGGGACTTTACTCACAACTAAACAAAAACGACGTTAGTGGTGCAACATTTACTCCAACAA

CTGTTGCAAACAGTGTGGCAGCATTTTCAGGATTGGTTAACGTTTATTACGATATAGCGA

TTGAAGATATGCCTATCACTCCATACGTTGGTGTTGGTGTTGGTGCAGCATATATCAGCA

ATCCTTCAGAAGCTAGTGCAGTTAAAGATCAAAAAGGATTTGGTTTTGCTTATCAAGCAA

AAGCTGGTGTTAGTTATGATGTAACCCCAGAAATCAAACTCTTTGCTGGGGCTCGTTATT

TTGGTTCTTATGGTGCTAGTTTTAATAAAGAAGCAGTATCAGCTACTAAAGAGATCAATG

TCCTTTACAGCGCTGTTGGTGCAGAAGCTGGAGT

>Wol 07

CAAGAATTGACGGCATTGAATATAAAAAAGGAACCGAAGTTCATGATCCTTTAAAAGCAT

CTTTTATGGGTGGTGGTGCTGCATTTGGTTATAAAATGGACGATATCAGGGTTGATGTTG

AGGGACTTTACTCACAACTAAACAAAAACGACGTTAGTGGTGCAACATTTACTCCAACAA

CTGTTGCAAACAGTGTGGCAGCATTTTCAGGATTGGTTAACGTTTATTACGATATAGCGA

TTGAAGATATGCCTATCACTCCATACGTTGGTGTTGGTGTTGGTGCAGCATATATCAGCA

ATCCTTCAGAAGCTAGTGCAGTTAAAGATCAAAAAGGATTTGGTTTTGCTTATCAAGCAA

AAGCTGGTGTTAGTTATGATGTAACCCCAGAAATCAAACTCTTTGCTGGTGCTCGTTATT

TTGGTTCTTATGGTGCTAGTTTTAATAAAGAAGCAGTATCAGCTACTAAAGAGATCAATG

TCCTTTACAGCGCTGTTGGTGCAGAAGCTGGAGT

>Wol 08

CAAGAATTGACGGCATTGAATATAAAAAAGGAACCGAAGTTCATGATCCTTTAAAAGCAT

CTTTTATGGCTGGTGGTGCTGCATTTGGTTATAAAATGGACGATATCAGGGTTGATGTTG

AGGGACTTTACTCACAACTAAACAAAAACGACGTTAGTGGTGCAACATTTACTCCAACAA

CTGGTGCAAACAGTGGGGCAGCATTTTCAGGATTGGTTAACGTTTATTACGATATAGCGA

TTGAAGATATGCCTATCACTCCATACGTTGGTGTTGGTGTTGGTGCAGCATATATCAGCA

ATCCTTCAGAAGCTAGTGCAGTTAAAGATCAAAAAGGATTTGGTTTTGCTTATCAAGCAA

AAGCTGGTGTTAGTTATGATGTAACCCCAGAAATCAAACTCTTTGCTGGTGCTCGTTATT

TTGGTTCTTATGGTGCTAGTTTTAATAAAGAAGCAGTATCAGCTACTAAAGAGATCAATG

TCCTTTACAGCGCTGTTGGTGCAGAAGCTGGAGT

>Wol 09

CAAGAATTGACGGCATTGAATATAAAAAAGGAACCGAAGTTCATGATCCTTTAAAAGCAT

CTTTTATGGCTGGTGGTGCTGCATTTGGTTATAAAATGGACAATATCAGGGTTGATGTTG

AGGGACTTTACTCACAACTAAACAAAAACGACGTTAGTGGTGCAACATTTACTCCAACAA

CTGTTGCAAACAGTGTGGCAGCATTTTCAGGATTGGTTAACGTTTATTACGATATAGCGA

TTGAAGATATGCCTATCACTCCATACGTTGGTGTTGGTGTTGGTGCAGCATATATCAGCA

ATCCTTCAGAAGCTAGTGCAGTTAAAGATCAAAAAGGATTTGGTTTTGCTTATCAAGCAA

AAGCTGGTGTTAGTTATGATGTAACCCCAGAAATCAAACTCTTTGCTGGTGCTCGTTATT

TTGGTTCTTATGGTGCTAGTTTTAATAAAGAAGCAGTATCAGCTACTAAAGAGATCAATG

TCCTTTACAGCGCTGTTGGTGCAGAAGCTGGAGT

>Wol 10

CAAGAATTGACGGCATTGAATATAAAAAAGGAACCGAAGTTCATGATCCTTTAAAAGCAT

CTTTTATGGCTGGTGGTGCTGCATTTGGTTATAAAATGGACGATATCAGGGTTGATGTTG

AGGGACTTTACTCACAACTAAACAAAAACGACGTTAGTGGTGCAACATTTACTCCAACAA

CTGTTGCAAACAGTGTGGCAGCATTTTCAGGATTGGTTAACGTTTATTACGATATAGCGA

TTGAAGATATGCCTATCACTCCATACGTTGGTGTTGGTGTTGGTGCAGCATATATCAGCA

ATCCTTCAGAAGCTAGTGCAGTTAAAGATCAAAAAGGATTTGGTTTTGCTTATCAAGCAA

AAGCTGGTGTTAGTTATGATGTAACCCCAAAAATCAAACTCTTTGCTGGTGCTCGTTATT

TTGGTTCTTATGGTGCTAGTTTTAATAAAGAAGCAGTATCAGCTACTAAAGAGATCAATG

TCCTTTACAGCGCTGTTGGTGCAGAAGCTGGAGT

>Wol 11

CAAGAATTGACGGCATTGAATATAAAAAAGGAACCGAAGTTCATGATCCTTTAAAAGCAT

CTTTTATGGCTGGTGGTGCTGCATTTGGTTATAAAATGGACGATATCAGGGTTGATGTTG

AGGGACTTTACTCACAACTAAACAAAAACGACGTTAGTGGTGCAACATTTACTCCAACAA

CTGTTGCAAACAGTGTGGCAGCATTTTCAGGATTGGTTAACGTTTATTACGATATAGCGA

TTGAAGATATGCCTATCACTCCATACGTTGGTGTTGGTGTTGGTGCAGCATATATCAGCA

ATCCTTCAGAAGCTAGTGCAGTTAAAGATCAAAAAGGATTTGGTTTTGCTTATCAAGCAA

AAGCTGGTGTTAGTTATGATGTAACCCCAGAAATCAAACTCTTTGCTGGTGCTCGTTATT

TTGATTCTTATGGTGCTAGTTTTAATAAAGAAGCAGTATCAGCTACTAAAGAGATCAATG

TCCTTTACAGCGCTGTTGGTGCAGAAGCTGGAGT

>Wol 12

CAAGAATTGACGGCATTGAATATAAAAAAGGAACCGAAGTTCATGATCCTTTAAAAGCAT

CTTTTATGGGTGGTGGTGCTGCATTTGGTTATAAAATGGACGATATCAGGGTTGATGTTG

AGGGACTTTACTCACAACTAAACAAAAACGACGTTAGTGGTGCAACATTTACTCCAACAA

CTGTTGCAAACAGTGTGGCAGCATTTTCAGGATTGGTTAACGTTTATTACGATATAGCGA

TTGAAGATATGCCTATCACTCCATACGTTGGTGTTGGTGTTGGTGCAGCATATATCAGCA

ATCCTTCAGAAGCTAGTGCAGTTAAAGATCAAAAAGGATTTGGTTTTGCTTATCAAGCAA

AAGCTGGTGTTAGTTATGATGTAACCCCAAAAATCAAACTCTTTGCTGGTGCTCGTTATT

TTGGTTCTTATGGTGCTAGTTTTAATAAAGAAGCAGTATCAGCTACTAAAGAGATCAATG

TCCTTTACAGCGCTGTTGGTGCAGAAGCTGGAGT

>Wol 13

CAAGAATTGACGGCATTGAATATAAAAAAGGAACCGAAGTTCATGATCCTTTAAAAGCAT

CTTTTATGGCTGGGGGTGCTGCATTTGGTTATAAAATGGACGATATCAGGGTTGATGTTG

AGGGACTTTACTCACAACTAAACAAAAACGACGTTAGTGGTGCAACATTTACTCCAACAA

CTGTTGCAAACAGTGTGGCAGCATTTTCAGGATTGGTTAACGTTTATTACGATATAGCGA

TTGAAGATATGCCTATCACTCCATACGTTGGTGTTGGTGTTGGTGCAGCATATATCAGCA

ATCCTTCAGAAGCTAGTGCAGTTAAAGATCAAAAAGGATTTGGTTTTGCTTATCAAGCAA

AAGCTGGTGTTAGTTATGATGTAACCCCAGAAATCAAACTCTTTGCTGGTGCTCGTTATT

TTGGTTCTTATGGTGCTAGTTTTAATAAAGAAGCAGTATCAGCTACTAAAGAGATCAATG

TCCTTTACAGCGCTGTTGGTGCAGAAGCTGGAGT

>Wol 14

CAAGAATTGACGGCATTGAATATAAAAAAGGAACCGAAGTTCATGATCCTTTAAAAGCAT

CTTTTATGGCTGGGGGTGCTGCATTTGGTTATAAAATGGACGATATCAGGGTTGATGTTG

AGGGACTTTACTCACAACTAAACAAAAACGACGTTAGTGGTGCAACATTTACTCCAACAA

CTGGTGCAAACAGTGTGGCAGCATTTTCAGGATTGGTTAACGTTTATTACGATATAGCGA

TTGAAGATATGCCTATCACTCCATACGTTGGTGTTGGTGTTGGTGCAGCATATATCAGCA

ATCCTTCAGAAGCTAGTGCAGTTAAAGATCAAAAAGGATTTGGTTTTGCTTATCAAGCAA

AAGCTGGTGTTAGTTATGATGTAACCCCAGAAATCAAACTCTTTGCTGGTGCTCGTTATT

TTGGTTCTTATGGTGCTAGTTTTAATAAAGAAGCAGTATCAGCTACTAAAGAGATCAATG

TCCTTTACAGCGCTGTTGGTGCAGAAGCTGGAGT

>Wol 15

CAAGAATTGACGGCATTGAATATAAAAAAGGAACCGAAGTTCATGATCCTTTAAAAGCAT

CTTTTATGGCTGGGGGGGCTGCATTTGGTTATAAAATGGACGATATCAGGGTTGATGTTG

AGGGACTTTACTCACAACTAAACAAAAACGACGTTAGTGGTGCAACATTTACTCCAACAA

CTGGTGCAAACAGTGTGGCAGCATTTTCAGGATTGGTTAACGTTTATTACGATATAGCGA

TTGAAGATATGCCTATCACTCCATACGTTGGTGTTGGTGTTGGTGCAGCATATATCAGCA

ATCCTTCAGAAGCTAGTGCAGTTAAAGATCAAAAAGGATTTGGTTTTGCTTATCAAGCAA

AAGCTGGTGTTAGTTATGATGTAACCCCAGAAATCAAACTCTTTGCTGGTGCTCGTTATT

TTGGTTCTTATGGTGCTAGTTTTAATAAAGAAGCAGTATCAGCTACTAAAGAGATCAATG

TCCTTTACAGCGCTGTTGGTGCAGAAGCTGGAGT
