# Supplementary figures and images for: Wolbachia and mosquitoes: Exploring transmission modes and coevolutionary dynamics in Shandong Province, China
Source: PLoS Negl Trop Dis. 2024 Sep 12;18(9):e0011944. doi: 10.1371/journal.pntd.0011944 (PMC11421781; doi:10.1371/journal.pntd.0011944)

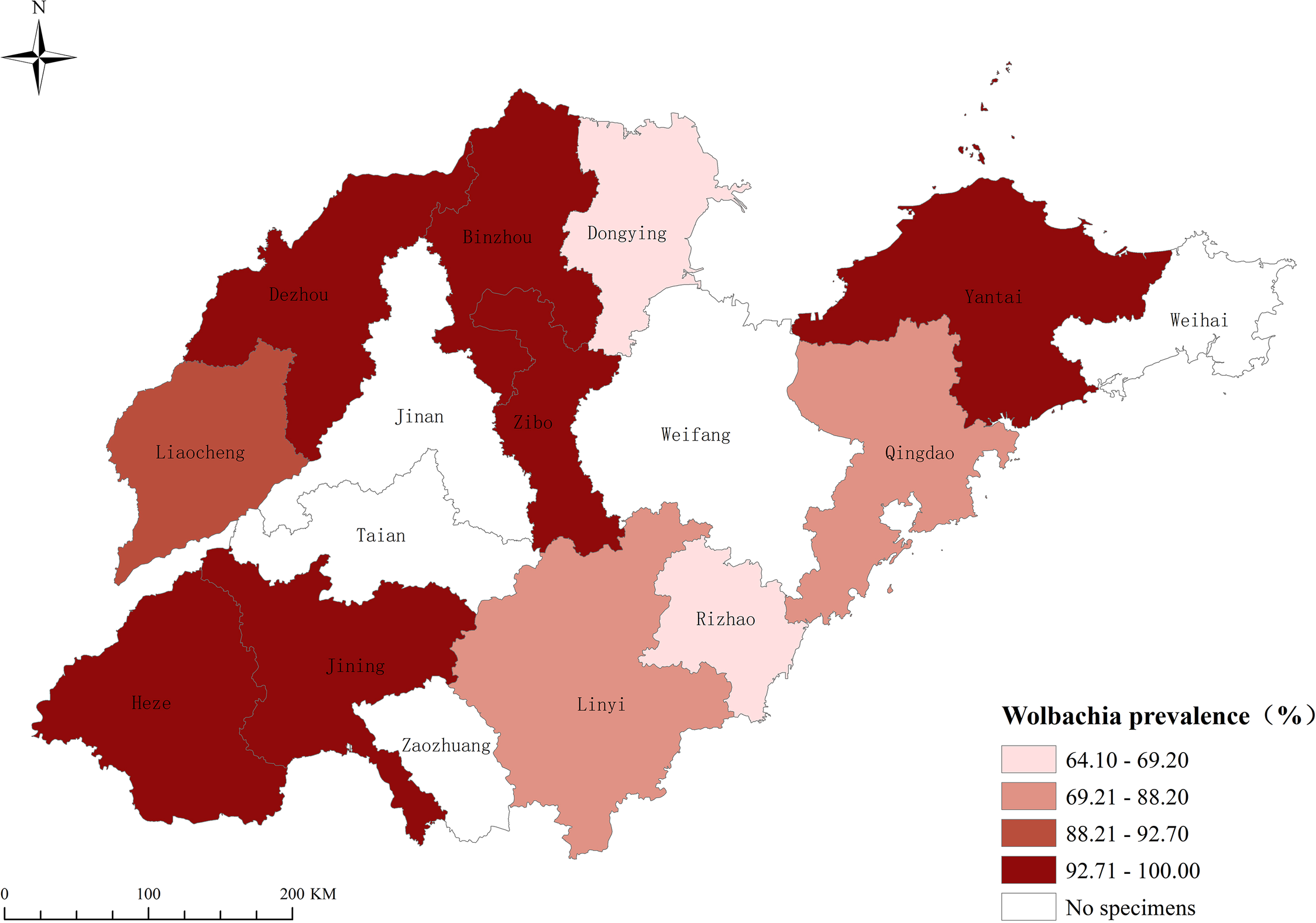

Supplement: S1 Fig — The prevalence in Rizhao and Dongying was 64.10%-69.20%; in Qingdao and Linyi, 69.21%-88.20%; in Liaocheng, 88.21%-92.70%; and in Dezhou, Yantai, Heze, Binzhou, Zibo and Jining, 92.715%-100.00%. The base layer of the map is from the Resource and Environment Science and Data Center (https://www.resdc.cn/). (TIF) [file pntd.0011944.s002.tif]

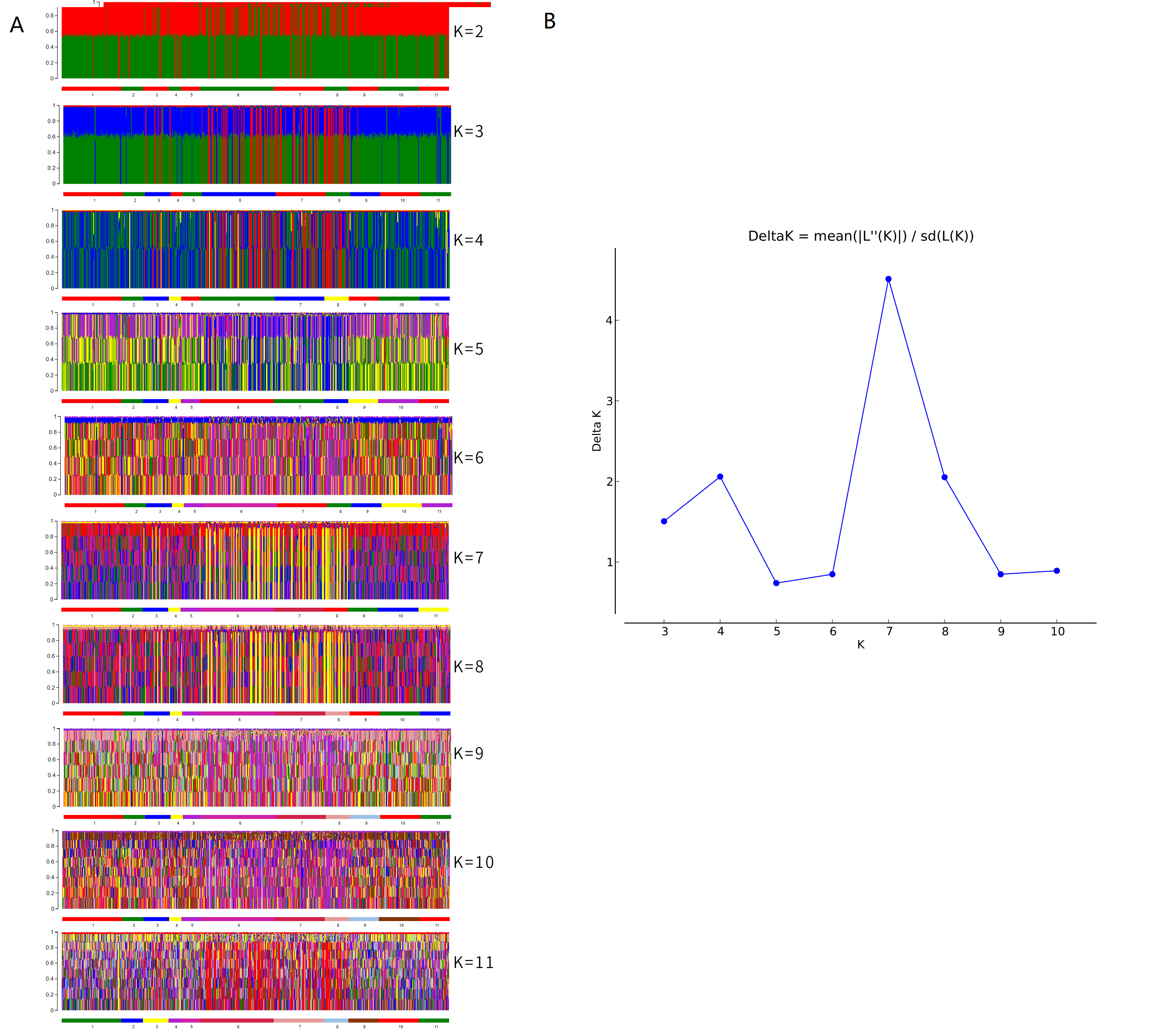

Supplement: S2 Fig — A. Stacked bar plots of STRUCTURE for K = 2 to 11 subgroups. Each individual is represented by a vertical bar, partitioned into coloured segments with the length of each segment representing the proportion of the individual’s genome; B. Delta K plotted against putative K ranging from 2 to 11. (TIF) [file pntd.0011944.s003.tif]

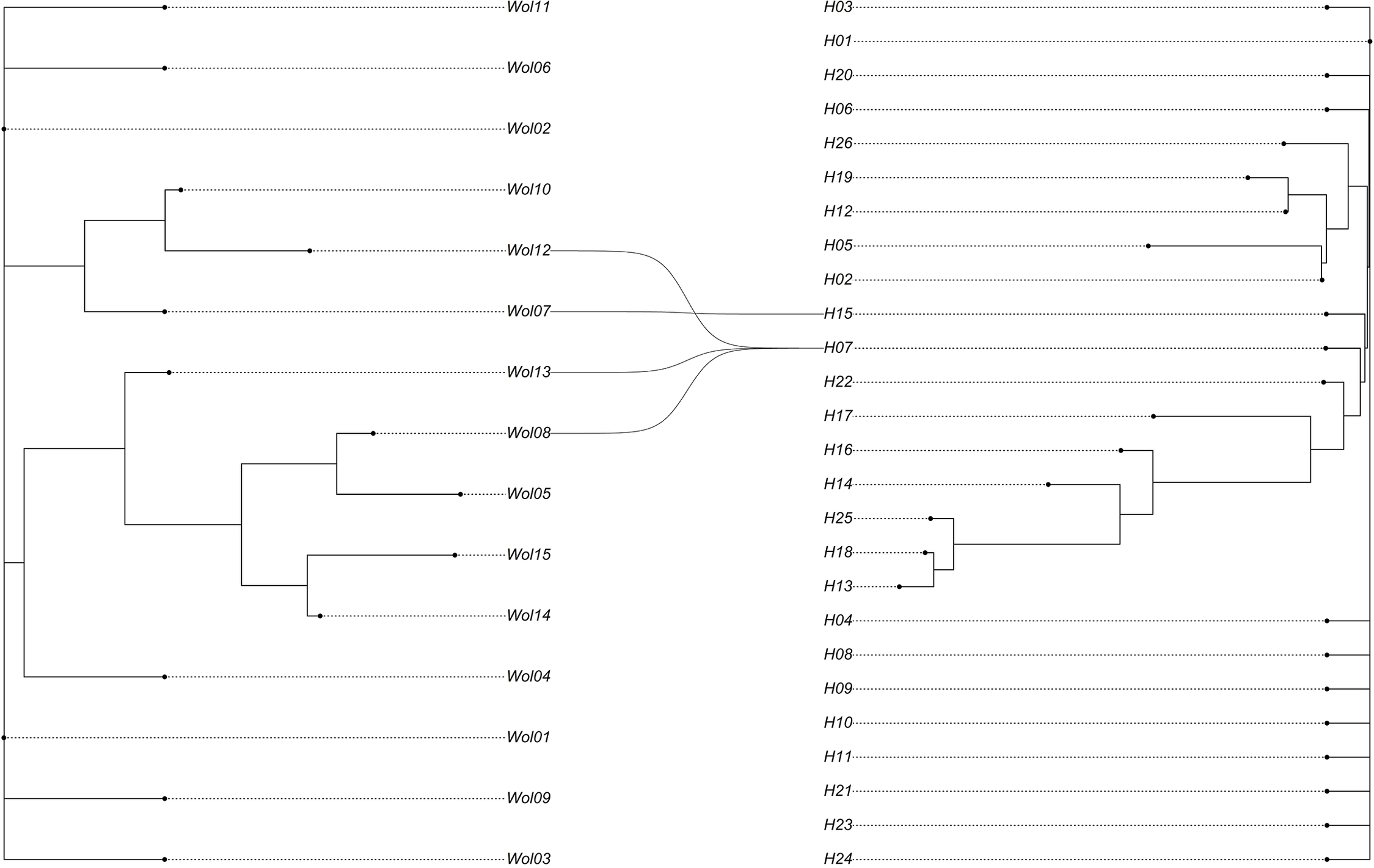

Supplement: S3 Fig — Lines indicate the host–endosymbiont association that was significant in the Global ParaFit test of congruence between host and endosymbiont phylogenies. (TIF) [file pntd.0011944.s004.tif]
